# Using mHealth to Provide Mobile App Users With Visualization of Health Checkup Data and Educational Videos on Lifestyle-Related Diseases: Methodological Framework for Content Development

**DOI:** 10.2196/20982

**Published:** 2020-10-21

**Authors:** Azusa Aida, Thomas Svensson, Akiko Kishi Svensson, Hirokazu Urushiyama, Kazuya Okushin, Gaku Oguri, Naoto Kubota, Kazuhiko Koike, Masaomi Nangaku, Takashi Kadowaki, Toshimasa Yamauchi, Ung-Il Chung

**Affiliations:** 1 Precision Health Department of Bioengineering, Graduate School of Engineering The University of Tokyo Tokyo Japan; 2 Department of Diabetes and Metabolic Diseases Graduate School of Medicine The University of Tokyo Tokyo Japan; 3 Department of Clinical Sciences Lund University Malmö Sweden; 4 School of Health Innovation Kanagawa University of Human Services Tonomachi Japan; 5 Department of Respiratory Medicine Graduate School of Medicine The University of Tokyo Tokyo Japan; 6 Department of Gastroenterology Graduate School of Medicine The University of Tokyo Tokyo Japan; 7 Department of Cardiology Graduate School of Medicine The University of Tokyo Tokyo Japan; 8 Department of Nephrology and Endocrinology Graduate School of Medicine The University of Tokyo Tokyo Japan; 9 Toranomon Hospital Tokyo Japan; 10 Clinical Biotechnology, Center for Disease Biology and Integrative Medicine Graduate School of Medicine The University of Tokyo Tokyo Japan

**Keywords:** apps, educational videos, health checkup, lifestyle-related disease, mHealth, prevention, telehealth, visualization

## Abstract

**Background:**

The number of people with lifestyle-related diseases continues to increase worldwide. Improving lifestyle behavior with health literacy may be the key to address lifestyle-related diseases. The delivery of educational videos using mobile health (mHealth) services can replace the conventional way of educating individuals, and visualization can replace the provision of health checkup data.

**Objective:**

This paper aimed to describe the development of educational content for MIRAMED, a mobile app aimed at improving users’ lifestyle behaviors and health literacy for lifestyle-related diseases.

**Methods:**

All videos were based on a single unified framework to provide users with a consistent flow of information. The framework was later turned into a storyboard. The final video contents were created based on this storyboard and further discussions with leading experts and specialist physicians on effective communication with app users about lifestyle-related diseases.

**Results:**

The app uses visualization of personal health checkup data and educational videos on lifestyle-related diseases based on the current health guidelines, scientific evidence, and expert opinions of leading specialist physicians in the respective fields. A total of 8 videos were created for specific lifestyle-related diseases affecting 8 organs: (1) brain–cerebrovascular disorder, (2) eyes–diabetic retinopathy, (3) lungs–chronic obstructive pulmonary disease, (4) heart–ischemic heart disease, (5) liver–fatty liver, (6) kidneys–chronic kidney disease (diabetic kidney disease), (7) blood vessels–peripheral arterial disease, and (8) nerves–diabetic neuropathy.

**Conclusions:**

Providing enhanced mHealth education using novel digital technologies to visualize conventional health checkup data and lifestyle-related diseases is an innovative strategy. Future studies to evaluate the efficacy of the developed content are planned.

## Introduction

Measures against noncommunicable diseases, such as cancer, heart disease, and stroke have been strengthened globally [1]. Above all, diabetes, evolving from metabolic syndrome, causes complications, which adversely impact a person’s health and overall quality of life and add to the medical expenditures on both individual and societal levels [1]. In Japan, the number of individuals with lifestyle-related diseases (eg, cancer, heart disease, and cerebrovascular disease) has been increasing. Lifestyle-related diseases account for 60% of deaths in Japan, and national medical expenses continue to increase at the rate of 1 trillion yen per year [2-4].

In Japan, annual health checkups are stipulated by various laws of the Ministry of Health, Labour and Welfare, such as the Industrial Safety and Health Act and the Health Promotion Act [5]. Specific health checkups and specific health guidance started in 2008. The targets of these health checkups and health guidance are people aged 40-74 years. As per the Elderly Medical Care Act, insurers must provide annual health checkups to those who are insured [5]. The aim is to identify people with metabolic syndrome and a subsequent risk of developing lifestyle-related diseases, such as diabetes, hypertension, and hyperlipidemia due to visceral fat accumulation [6,7]. Unlike diseases treated only with medication, the key to managing metabolic syndrome and lifestyle-related diseases is to modify lifestyle behavior. Achieving such behavioral change is a challenge, as behavior in any target population differs by age, sex, occupation, lifestyle factors (eg, smoking, alcohol consumption, exercise, and diet), individual background, knowledge, and understanding of health issues that have accumulated over many years [7,8].

Although the impact of risky lifestyle behaviors on health has long been established, the importance of health literacy is now increasingly recognized [8,9]; and the association between health literacy and lifestyle behaviors has been widely confirmed [8,10]. Health literacy can be understood by using lay terms related to the ability of individuals to address health issues in a complex society. However, the rapid growth in its recognition has led to multiple interpretations of the concept, which may cause confusion [8]. Sørensen et al [11] defined health literacy as “people’s knowledge, motivation and competences to access, understand, appraise, and apply health information in order to make judgments and take decisions in everyday life concerning healthcare, disease prevention and health promotion to maintain or improve quality of life during the life course.” Moreover, Berkman et al [12] defined health literacy by emphasizing similar abilities or “know-how” that could be used to “communicate about” issues related to health.

Improved health literacy about lifestyle-related diseases may thus lead to improved health behaviors, which in turn are important for sustainable prevention of lifestyle-related diseases. One route that could prove beneficial for improving health literacy is the use of mobile health (mHealth) apps.

In recent years, information and communications technologies (ICTs) have advanced rapidly, and the number of mHealth apps has been increasing [13]. mHealth is an expanding area within eHealth, which includes medical and public health information services provided via the internet and related technologies [14]. mHealth allows the general public to gain access to health advice or behavioral interventions. In Japan, the provision to use ICT for specific health guidance was initially announced in 2013 [15] and revised in 2018 [16]. Specific health guidance utilizing ICTs has the potential to increase participation rates due to its convenience to remote users as well as busy working professionals, allowing effective health guidance without in-person meetings.

Indeed, the delivery of educational content through videos can replace the current use of in-person communication in providing health checkup data and information about lifestyle-related diseases that may be difficult to understand for those who are not health care professionals [13,17]. With the expansion of mHealth services, the range of methods available to improve users’ health literacy is increasing (eg, use of apps with videos, photos, and SMS text messages) [13,18]. To our knowledge, however, no app has yet been developed that encourages healthy behavior among persons with metabolic syndrome and high risk of lifestyle-related diseases, which (1) uses visualization of health checkup data to describe possible future lifestyle-related disease and (2) provides evidence-based educational content to improve health literacy about relevant lifestyle-related diseases.

MIRAMED, the lifestyle intervention app described in this paper, aims to be compatible with the Japanese Ministry of Health, Labour and Welfare’s summary [19] of the present health promotion guidelines. In this paper, we describe the development of the visual content of the app that aims to improve user health literacy and lifestyle-related behaviors by converting the individual’s numerical health checkup data into easy-to-understand visual information. We focus on the process of creating educational videos for this app based on the current guidelines and expert opinions from specialist physicians.

## Methods

### Development of Educational Contents

**Figure 1:**
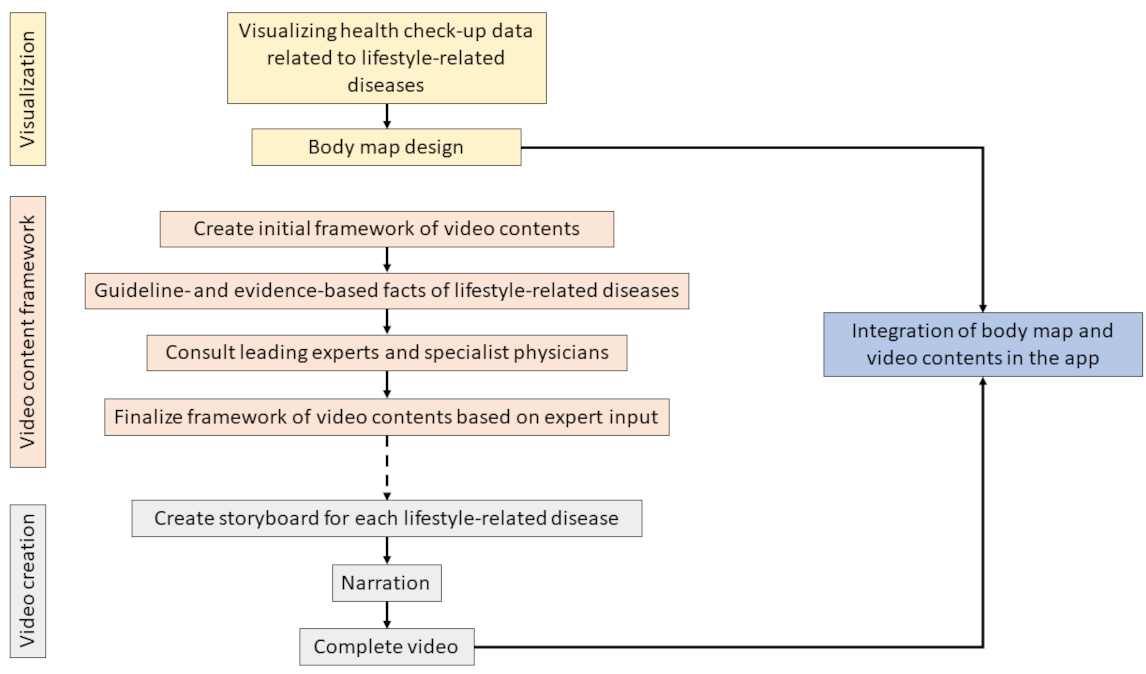
Flowchart of the developmental process of the body map (visualization of health checkup data) and educational videos used in the lifestyle intervention app MIRAMED.

The target audiences of the app were people with metabolic syndrome and high risk of metabolic syndrome. The first step in the app’s lifestyle intervention process was raising users’ awareness of the current status of their health and potential risks of lifestyle-related diseases by visualizing their annual health checkup data. This step was facilitated by using icons displaying key organs in combination with educational videos that describe typical lifestyle-related diseases of those organs. Subsequently, users were prompted to set personal goals for lifestyle change in 5 key lifestyle categories: nutrition, smoking and alcohol, exercise, sleep, and stress. An intervention period of 3 months was set based on the user’s understanding of their baseline health status. The user then received daily evaluations related to the lifestyle categories and weekly feedback on their progress. This paper focuses on the process of creating the visualization of health checkup data and the creation of the educational videos used in the MIRAMED app. The overview of this process is illustrated in Figure 1.

The MIRAMED app and its contents were developed by the Precision Health group, Center of Innovation at the University of Tokyo in Japan; and the study was approved by the ethics committee of the Department of Bioengineering at the University of Tokyo (approval number: KE18-44). This research was supported by the Center of Innovation Program from Japan Science and Technology Agency. The funding agency had no role in the design of the study; collection, analysis, and interpretation of data; writing of the report; and decision to submit the paper for publication.

### Visualization of Health Checkup Data

**Figure 2:**
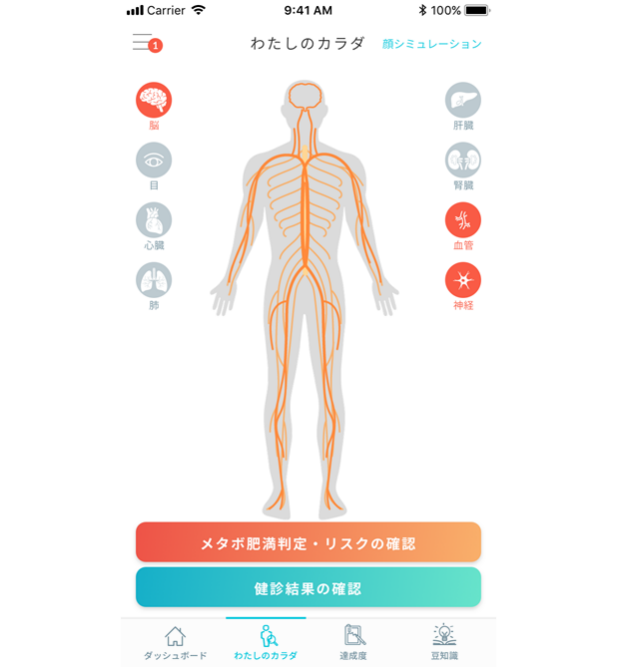
User interface of the app showing a body map with the visualization of a user’s risks of lifestyle-related diseases.

Annual health checkup data were visualized in a body map using icons of 8 organs related to lifestyle-related diseases: brain, eyes, lungs, heart, liver, kidneys, blood vessels, and nerves. When a user’s health checkup data indicated possible risks associated with a potentially affected organ, the icons were rendered in orange coloring (Figure 2).

### Creation of Educational Videos About Lifestyle-Related Diseases

The purpose of educational videos in the app was to improve users’ health literacy on potential health risks indicated by their health checkup data. In accordance with the Japanese Ministry of Health, Labour and Welfare’s summary [19,20] of the present health promotion guidelines in Japan, 8 videos were created. Each video matched with 1 of the 8 selected organs and provided content on a lifestyle-related disease relevant to that organ. First, a unified framework was developed on which all the videos were based. The aim of this framework was to make users familiar with the flow of information in each video and to create cohesiveness across videos covering different lifestyle-related diseases. A framework was then developed into health condition–specific storyboards, which were in turn made into educational videos. Discussions were held with the leading experts and specialist physicians in the fields of neurology, diabetes and metabolic diseases, respiratory medicine, cardiology, gastroenterology, nephrology, and endocrinology. These experts and physicians consulted on how to convey evidence-based and health guidelines–compliant message for each disease to the app users. It was agreed that the unified framework for the videos would contain the following 5 sections (Table 1): disease name, explanation of the disease, symptoms, important facts, and improvement and prevention of the disease. It was required that the contents of each section were grounded in the scientific literature. Owing to the limitations on playback time of each section, word count for the explanation of each section was also restricted. The framework for all the videos was shared with the production company, and visual designers were provided with instructions on the appropriate animated contents for each section. Extensive discussions were held regarding the comprehensibility of each section from a user’s point of view. This feedback allowed for the creation of storyboards, which were discussed further before developing the final product.

**Table 1:** The framework for “Diabetic neuropathy.”

Sections	Explanation
Disease name	Diabetic neuropathy
Explanation of the disease	The systemic peripheral nervous system is damaged due to chronic hyperglycemia. Development of the condition involves peripheral nerve metabolism abnormality, decreased chronic blood flow in nerve tissue due to microangiopathy, and hypoxia. The pathogenic mechanism emphasizes polyol pathway enhancement, free radicals, abnormal lipid metabolism, protein glycation, and inflammatory factors [21]
Symptoms	In a typical example, signs appear bilaterally in the toes and sole [21,22] The signs expand to more proximal parts, such as ankles and lower legs. As the condition progresses, hands start showing signs in a glove-sock–like pattern [21,22] Sensory nerves, motor nerves, and autonomic nerves are damaged, in that order [21-23] Autonomic neuropathy can involve all systems in the body. It causes a substantial increase in morbidity and mortality, especially in the presence of cardiovascular autonomic neuropathy [21-24]
Important facts	Diabetes causes various metabolic disorders centered on persistent hyperglycemia due to insufficient action of insulin. This impairment occurs in the order of neuropathy, retinopathy, and nephropathy [21,25]
Improvement and prevention	Blood glucose control from an early stage [21,25-28]

**Figure 3:**
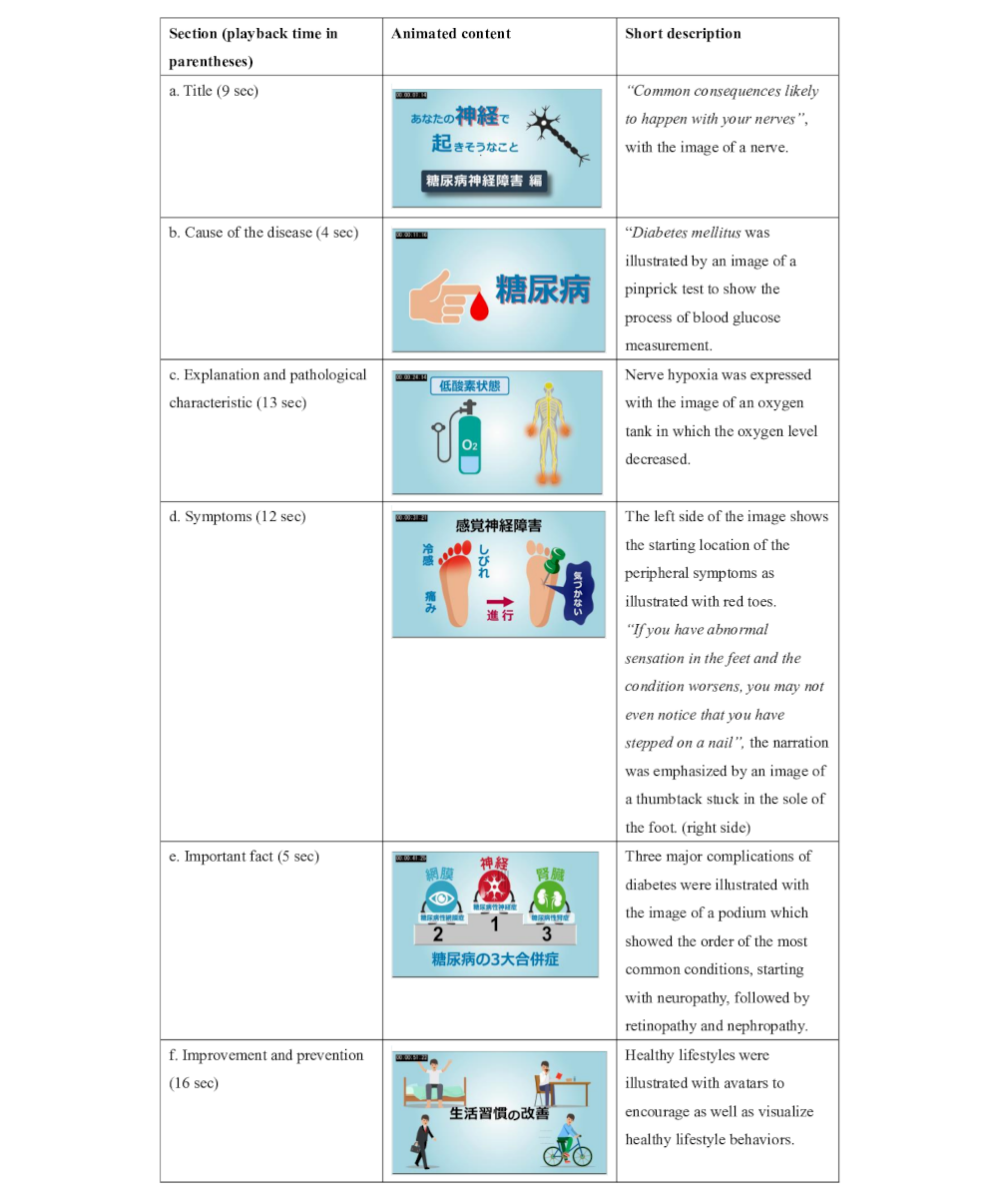
The storyboard of diabetic neuropathy.

Figure 3 gives an example of the storyboard, which was later turned into the educational video for “Diabetic neuropathy,” a lifestyle-related nerves disease (as indicated by the corresponding icon). The storyboard additionally contained a short description of each section and the maximum length in minutes for the corresponding narration.

## Results

A total of 8 videos were created by matching an organ with its associated lifestyle-related disease: (1) brain–cerebrovascular disorder, (2) eyes–diabetic retinopathy, (3) lungs–chronic obstructive pulmonary disease, (4) heart–ischemic heart disease, (5) liver–fatty liver, (6) kidneys–chronic kidney disease (diabetic kidney disease), (7) blood vessels–peripheral arterial disease, and (8) nerves–diabetic neuropathy. Each of the videos had a playback time of around 1 minute to retain the user’s attention on the contents of the video.

In the example shown in Figure 2, the app indicates that the user had 3 potentially affected organs (brain, blood vessels, and nerves), which are rendered in orange coloring. Upon touching the icon of the glowing organ, the user would be taken to the educational video about the most common lifestyle-related diseases associated with that organ. A detailed description is provided for diabetic neuropathy.

The total playback time of the educational video for diabetic neuropathy was 59 seconds. The title was purposefully chosen as a statement, “Common consequences likely to happen with your nerves,” illustrated with the image of a nerve (Figure 3A). The title was seamlessly connected with a narrated explanation of the underlying cause of the condition: “The main cause of diabetic neuropathy is diabetes mellitus” [21,23]. This was shown along with an image of a pinprick test, meant to illustrate the process of blood glucose measurement (Figure 3B). As a further explanation of disease pathology, the narration continued, “If your blood glucose level remains high, the blood vessels that nourish the nerves will have an accumulation of waste products. This results in damage to the blood vessels, which leads to hypoxia and impaired nerve function.” The nerve hypoxia was expressed with the image of an oxygen tank in which the oxygen level decreased (Figure 3C). This was complemented with the figure of a human with highlighted hands and feet to suggest that symptoms usually start in the peripheral extremities. Typical symptoms were described in the order in which they usually appear, starting with sensory nerves, followed by motor nerves, and finally autonomic nerves [21-23]. The early-stage sensory disorder was explained as starting with spontaneous pain, numbness, and abnormal sensation in the lower limbs at an early stage of the onset [21,22,24,29]. The starting location of the peripheral symptoms was illustrated with red toes (left side of Figure 3D). Narration explained late-stage sensory disorder to the user. “If you have abnormal sensation in the feet and the condition worsens, you may not even notice that you have stepped on a nail.” The narration was combined with an image of a thumbtack stuck in the sole of foot (right side of Figure 3D).

The important fact section highlighted the following 3 major complications of diabetes: (1) neurological disorder–neuropathy, (2) eye disorder–retinopathy, and (3) kidney disorder–nephropathy [21,25]. The image of a podium (Figure 3E) illustrated the order of the most common conditions, starting with neuropathy, followed by retinopathy and nephropathy [21,25]. Finally, for the prevention or improvement of the condition, several factors were considered, such as proper diet (managing calory intake [21,25,28], starting each meal with vegetables [25], not eating sweets [25,28], eating breakfast every morning [25,28]); increased physical activity (combining aerobic and anaerobic exercises) [25,28]; and reducing stress in daily life [28]. In the video, healthy lifestyles were illustrated with avatars to encourage and visualize healthy lifestyle behaviors, such as waking up early in the morning, commuting on foot, bicycling, and allowing oneself time to relax (eg, by reading books) (Figure 3F).

## Discussion

The purpose of the MIRAMED app is to improve users’ health literacy and encourage lifestyle-related behaviors through the personalization of health and lifestyle information. This occurs through the following means: (1) visualization of users’ annual health checkup data using icons that feature potential affected organs along with the possible lifestyle-related diseases of the featured organs, and (2) provide educational content on lifestyle-related diseases through videos. Given that informed, active, motivated, knowledgeable, and confident users can improve their health outcomes [30] and reduce medical costs [31], the app’s content may support a change of health behavior in users. To our knowledge, this is the first study to describe potential lifestyle-related diseases by visualizing users’ personal annual health checkup data in combination with educational videos to describe typical lifestyle-related diseases based on the scientific literature and opinions of leading experts and specialist physicians.

Improvement in self-efficacy significantly increases the likelihood that a health intervention (eg, weight loss [32,33] or smoking cessation [34]) will be successfully maintained. Learning and using medical terminology resembles learning a new language, and this is one reason why non–health care professionals face difficulty in understanding health checkup data and lifestyle-related diseases [35]. Visual and auditory augmentation of printed medical terms promotes the understanding of individual health status and provides an opportunity for repeated engagement in learning sessions [35].

App users can access educational videos about typical lifestyle-related diseases by touching icons of the respective organs. The purpose of these videos was to contribute to the improvement of the user’s health literacy. All videos were developed according to a consistent and unified framework. The framework was created in order to provide the user with a familiar structure and consistency across all videos, thus making the information from a wide range of medical specialties easily accessible. The playback time of each video was limited to around 1 minute, and the narration was conducted by the same narrator across all the videos and information. Moreover, both developers and specialists agreed that the contents of the app could be easily understood, even by those without a medical background.

The global penetration of mobile phones is growing rapidly, with up to 90% penetration in countries with high-income economies [36]. For those with a lifestyle-related disease or other chronic health problems, especially individuals living in rural areas who do not have access to medical services by other means, knowledge applicable to daily life is essential for effective self-management of lifestyle-related diseases [35].

This development had a few limitations worth noting. First, the content of the app was developed for the Japanese public. Thus, all the content was written in Japanese. However, it is possible to translate the app and its content into other languages. Second, information on how many times a user played each video and the number of videos watched by a user was not collected. Third, in general, there is a lack of standardized measurement tools to assess health literacy and conceptual framework for health literacy [8,37,38]. However, the future versions of the app can include measures of health literacy, for example, the Rapid Estimate of Adult Literacy in Medicine, Test of Functional Health Literacy in Adults (TOFHLA), or Newest Vital Sign [37-39]. We are presently investigating user experience with the usability of the app; accessibility of the contents; and changes in user lifestyle behaviors, weight, and waist circumference.

In conclusion, this study described the development of visually enhanced health education materials using new digital technologies with an innovative strategy to visualize conventional health checkup data. Although developed as educational videos for lifestyle-related diseases, these contents are expected to be used in various fields. Further evaluation of the effectiveness of the developed contents is needed.

## Abbreviations

ICT: information and communications technology
mHealth: mobile health
TOFHLA: Test of Functional Health Literacy in Adults
